# Characterization of epithelial cells, connective tissue cells and immune cells in human upper airway mucosa by immunofluorescence multichannel image cytometry: a pilot study

**DOI:** 10.1007/s00418-020-01945-y

**Published:** 2020-11-29

**Authors:** Aris I. Giotakis, Jozsef Dudas, Rudolf Glueckert, Daniel Dejaco, Julia Ingruber, Felix Fleischer, Veronika Innerhofer, Leyla Pinggera, Ljilja Bektic-Tadic, Sarah A. M. Gabriel, Herbert Riechelmann

**Affiliations:** 1 Department of Otorhinolaryngology, Medical University of Innsbruck, Innsbruck, Austria; 2 University Clinics Innsbruck, Tirol Kliniken, Anichstrasse 35, 6020 Innsbruck, Austria

**Keywords:** Image cytometry, Fluorescent antibody technique, Immunohistochemistry, Nasal mucosa, Sinusitis

## Abstract

Epithelial, connective tissue and immune cells contribute in various ways to the pathophysiology of chronic rhinosinusitis (CRS). However, data of their distribution in upper airway mucosa are sparse. We aimed to provide quantitative, purely informative data on the distribution of these cell lineages and their coexpression patterns, which might help identifying, e.g., cells in the epithelium undergoing through epithelial–mesenchymal transition (EMT). For this purpose, we used immunofluorescence multichannel image cytometry (IMIC). We examined fixed paraffin-embedded tissue samples (FFPE) of six patients with chronic rhinosinusitis (CRS) and of three patients without CRS (controls). The direct-conjugated antibodies pancytokeratin, vimentin and CD45/CD18 were used for coexpression analysis in epithelial layer and lamina propria. Image acquisition and analysis were performed with TissueFAXS and StrataQuest, respectively. To distinguish positive from negative expression, a ratio between cell-specific immunostaining intensity and background was developed. Isotype controls were used as negative controls. Per patient, a 4.5-mm^2^ tissue area was scanned and a median of 14,875 cells was recognized. The most common cell types were cytokeratin-single-positive (26%), vimentin-single-positive (13%) and CD45/CD18-single-positive with CD45/CD18–vimentin-double-positive cells (29%). In the patients with CRS, CD45/CD18-single-positive cells were 3–6 times higher compared to the control patients. In the epithelial layer, cytokeratin–vimentin-double-positive EMT cells were observed 3–5 times higher in the patients with CRS than in the control patients. This study provided quantitative data for the distribution of crucial cell types in CRS. Future studies may focus on the distribution and coexpression patterns of different immune cells in CRS or even cancer tissue.

**Electronic supplementary material:**

The online version of this article (10.1007/s00418-020-01945-y) contains supplementary material, which is available to authorized users.

## Introduction

Chronic rhinosinusitis (CRS) is a disabling disease affecting more than 10% of the general population (Hastan et al. 2011). CRS is classified into CRS with nasal polyps (CRSwNP) and CRS without nasal polyps (CRSsNP) (Fokkens et al. 2020). Epithelial cells, connective tissue cells and immune cells contribute in various ways to the pathogenesis of CRS. However, few data on the relative abundance of these cell lineages are available (Jiang et al. 2013). Our aim was to provide quantitative, purely informative data of the distribution of these cell lineages and their coexpression patterns in healthy and diseased upper airway mucosa. Coexpression patterns might identify, e.g., cells in the epithelium undergoing through epithelial–mesenchymal transition (EMT) (Li et al. 2019; Yan et al. 2019). We investigated whether immunofluorescence multichannel image cytometry (IMIC) provides such data.

Immunohistochemistry (IHC) and immunofluorescence (IF) microscopy are standard methods for cellular and molecular characterization (Polzehl et al. 2006), but quantitative analysis is not easy to perform. Flow cytometry provides quantification of different cells. Moreover, reliable analysis of biomarker coexpression using multiple fluorescence channels is possible (Beavis and Pennline 1994). Regardless, solid tissues must be transferred to single-cell suspensions for flow cytometry, which may result in the loss of cellular epitopes (Kirsche et al. 2010). Multicolor image cytometry provides high-throughput quantitative and objective data of formalin-fixed paraffin-embedded (FFPE) tissue samples (Ecker et al. 2006).

In recent years, we have developed four-channel IF microscopy for the simultaneous detection of three antibodies and the cell nucleus with 4′,6-diamidin-2-phenylindole (DAPI) in the mucosa of the upper airway. IMIC allows for quantification of various cellular subtypes and patterns of coexpression in FFPE tissue samples (Ecker and Steiner 2004; Ecker et al. 2006; Healy et al. 2018). For the detection of epithelial cells, connective tissue cells and immune cells, we used the epithelial marker pancytokeratin, the fibroblast marker vimentin and the leukocyte markers CD45/CD18. Epithelium and lamina propria (LP) were manually outlined and separately examined. We intended to detect (1) epithelial cells in epithelium, (2) glandular cells in LP, (3) connective tissue cells in LP, including fibroblasts and endothelial cells, (4) all types of leukocytes in epithelium and/or LP and (5) EMT cells in epithelium.

## Materials and methods

### Patient population

Patients with CRSsNP and CRSwNP (Fokkens et al. 2012) and patients undergoing surgical treatment of the inferior turbinate with no signs of CRS were included as controls (Zhang et al. 2008; Van Zele et al. 2006). Exclusion criteria were revision surgery and use of topical and/or systemic steroids in the 6 weeks preceding tissue sampling. Clinical data were available in the CRS database of the Department of Otorhinolaryngology, Medical University of Innsbruck. The study conforms to the standards of the Declaration of Helsinki. The Ethics Committee of the Medical University of Innsbruck approved the study (1228/2017), and informed consent was obtained from all subjects before sample collection. For this pilot study, we retrospectively selected nine patients from our CRS patient database. Of these, three had CRSsNP and three CRSwNP; three patients with inferior turbinate hypertrophy served as controls.

### Specimen harvest

Tissue samples of CRS and control patients were collected from the anterior ethmoidal region during endoscopic sinus surgery (ESS) and from resected inferior turbinate mucosa, respectively (Polzehl et al. 2006). The samples were transferred to Modified Eagle’s Medium with Earle’s Salts without L-glutamine (PAA Laboratories GmbH, Pasching, Germany) and stored at 2 °C for 30 min.

### Embedding and cutting

The samples were fixed in buffered 4% formaldehyde solution (SAV, Liquid Production GmbH, Flintsbach am Inn, Germany) overnight and kept in phosphate-buffered saline (PBS) supplied with 1 g/L sodium azide prior to embedding. Dehydration and paraffin impregnation were performed with a microwave tissue processor paraffin embedding system (Histos 5, Milestone, Bergamo, Italy) according to the manufacturer’s recommendations. Biopsies were sectioned to 5-µm thickness using an HM 355S microtome (Microm, Walldorf, Germany) and transferred to Superfrost Plus slides (Menzel, Braunschweig, Germany).

### Antigen retrieval, immunostaining and autofluorescence reduction

Colabeling of the epithelial marker pancytokeratin, the fibroblast marker vimentin and the leukocyte marker CD45/CD18 was achieved with a fully automated immunostaining system (Ventana Discovery classic, Roche, Mannheim, Germany) using direct-conjugated primary antibodies. The antibodies pancytokeratin, vimentin, CD45 and CD18 were directly coupled to the fluorescent dyes AlexaFluor 488, eFluor 570, AlexaFluor 594 (Biolegend, San Diego, USA) and AlexaFluor 594 (Bioss Antibodies, Woburn, USA), respectively. As a nuclear counterstain, we used 4′,6-diamidin-2-phenylindole (DAPI, 1:46.000, Thermo Fisher Scientific, Darmstadt, Germany) (Tarnowski et al. 1991). Antibody details are listed in Table 1.

**Table 1:** Characteristics of the antibodies

Epitope	Direct conjugate	Emission (nm)	Excitation (nm)	Expression	Host	Subtype	Dilution	Catalog number	Batch number	Clone	Supplier
Pancytokeratin	Alexa Fluor 488	515	488	Cytoplasmic	Mouse	IgG1	1:100	628,608	B261403	C-11	Biolegend^a^
Vimentin	eFluor 570	585	546	Cytoplasmic	Mouse monoclonal	IgG1	1:100	41–9897-82	4,315,949	V-9	Invitrogen^b^
CD45	Alexa Fluor 594	617	590	Cytoplasmic/cell membrane	Mouse monoclonal	IgG1	1:100	304,060	B221697	HI30	Biolegend^a^
CD18	Alexa Fluor 594	617	590	Cytoplasmic/cell membrane	Rabbit polyclonal	IgG	1:200	BS-3264R-A594	AG10119513	Polyclonal	Bioss^c^
Isotype control 1	Alexa Fluor 488	515	488	None	Mouse	IgG1	1:200	MA5-18,167	4,565,858	-	Invitrogen^b^
Isotype control 2	eFluor 570	585	546	None	Mouse	IgG1	1:100	41–4714-82	1,987,966	-	Invitrogen^b^
Isotype control 3	Alexa Fluor 594	617	590	None	Mouse	IgG1	1:100	400,174	B262874	-	Biolegend^a^

^a^San Diego, USA

^b^eBioscience, Life Technologies, Darmstadt, Germany

^c^Bioss Antibodies, Woburn, USA

The pancytokeratin antibody recognizes human cytokeratin 4, 5, 6, 8, 10, 13 and 18. This combination should cover all epithelial cells recognized in the nasal mucosal membrane (Stosiek et al. 1992; Liu et al. 2017; Hicks et al. 1995). Vimentin is expressed in fibrocytes, fibroblasts, endothelial cells, and leukocytes, including macrophages, neutrophils, lymphocytes (Satelli and Li 2011; Fuchs and Weber 1994; Steinert et al. 1993; McKeon et al. 1986; Steinert 1993; Crystal et al. 2008; Lilienbaum et al. 1986; Lan et al. 2018), and blood platelets (Ahmed et al. 2009). The V-9 clone recognizes endothelial cells, fibroblasts, smooth muscle cells and lymphoid cells. The CD45 antibody used is the HI30 monoclonal antibody, which recognizes all forms of human CD45. It reacts with lymphocytes, granulocytes, monocytes and eosinophils, but not with mature erythrocytes and platelets. CD18 plays an important role in neutrophil transmigration (Fukuda and Schmid-Schonbein 2003) and is expressed in all leukocytes.

After drying overnight and incubation at 60 °C for 1 h, microscopy slides were positioned on individually heated pads of the immunostainer. Deparaffination was accomplished with a model-specific detergent at 75 °C for 8 min (Ventana EZ Prep, 950–102) followed by heat-induced antigen retrieval by boiling individual slides in EDTA buffer (Cell Conditioning Solution CC1, Ventana 950–124). The sections were then incubated with the conjugated primary antibodies at 37 °C for 1 h. At all incubation steps, the 380–420-µl liquids were shielded with an oil film (Ventana liquid coverslip, LCS 650–010), counterstained with DAPI for 5 min, and washed with reaction buffer twice and with PBS once. To reduce autofluorescence, a commercial quenching kit (Vector TrueVIEW™ Autofluorescence Quenching Kit, SP-8400, Vector Laboratories, Burlingame, California, United States) was applied according to manufacturer recommendations, and the sample was mounted with an aqueous medium (Vectashield vibrance M, VECH-1700).

Isotype controls were used to differentiate specific fluorescence signals from background noise or nonspecific antibody binding (Table 1). To compensate for channel spillover, three tissue samples were stained with DAPI and single-immunostained (cytokeratin alone or vimentin alone or CD45/CD18 alone), and one sample was used as an isotype control.

### Image acquisition

For image acquisition, the TissueFAXS PLUS system (TissueGnostics, Vienna, Austria) was used. Images of the immunostained tissue samples were acquired with an apochromat 40×, 0.6 ‘air’ lens (serial number: 420660–9970, Zeiss, Jena, Germany) based on a Zeiss Axio Imager Z2 Microscope (serial number: 3523 0000 186) equipped with an 8-slide automatic stage (Märzhäuser, Wetzlar, Germany), magnification 400×. An X-Cite 120Q (Lumen Dynamics, Ontario, Canada) was used as the fluorescent light source of a proprietary 120-W mercury vapor short arc lamp with an output spectrum of 350–600 nm at a relative intensity of 60–100%.

The fluorescence microscope was equipped with four bandwidth filters to detect fluorescence of different wavelengths in four channels, which corresponded to fluorophores DAPI, AF488, eFluor 570 and AF594 (Table 2). The fluorescence intensity in the four channels was imaged sequentially and merged into one image. Colors were arbitrarily chosen for each channel: green was used for cytokeratin (AF488), red for vimentin (eFluor 570) and yellow for CD45/CD18 (AF594); blue was reserved for DAPI (DAPI).

**Table 2:** Fluorescence microscope filters and fluorophores used in the study

Zeiss filter set	Item number	Excitation filter (nm)	Beamsplitter (nm)	Emission filter (nm)	Fluorophore
44	000,000–1114-459	Band pass 475/40	500	Band pass 530/50	AF 488
20	488,020–9901-000	Band pass 546/12	560	Band pass 575—640	eFluor 570
49	488,049–9901-000	Glass 365	395	Band pass 445/50	DAPI
71	488,071–0000-000	Band pass 592/24	615	Band pass 675/100	AF 594

After a preview of whole slides with a 2.5 × lens, the software function ‘automatic tissue detection’ was applied. By default, the detection is run on the entire preview image; it can also run automatically on random parts of the whole slide. We chose to acquire parts of the whole slide to limit the illumination time of the fluorescent tissue samples. For explorative reasons, we chose detection of the whole slide for one patient. Severe variations between whole- and partial-slide acquisition samples would lead us to identify potential serious effects of illumination time to fluorescent signal.

Image tiles were acquired fully automatically with a 40 × air lens using extended depth of focus with autofocus and two 1.7-µm steps above and two below (five in total including middle autofocus), with a USB-connected PCO pixelfly CCD (serial number: 9070000310, PCO AG, Kelheim, Germany) monochrome camera in 14-bit dynamic range grayscale with 15% image overlap.

The exposure time was selected with the help of a range indicator reporting camera sensor over- or undersaturation for each pixel to avoid any loss of information (Dudas et al. 2018). Representative image tiles were automatically stitched. All parameters were saved and reused with each image acquisition to assure equal acquisition conditions. This resulted in acquisition of the tissue specimens in 14-bit dynamic range resolution. In a 10-mm^2^ tissue area, 238 image tiles (FOVs) were taken. One image tile is 1392 × 1040 pixels and 353.25 × 263.8 µm. One pixel is 253.78 nm. One image tile corresponds to 2.76 megabytes. For acquisition of a 10-mm^2^ tissue area, a scan of a 14.2-mm^2^ area is required. After acquisition, 30 min of manual focus correction of blurred areas was occasionally required for 10-mm^2^ tissue areas. To reduce the need for manual focus correction, tissue samples should be waste-free.

### Image analysis

We used the image analysis software StrataQuest (TissueGnostics) for image analysis with an HP Z 440 computer containing an Intel Xeon E5-1620 v3 quad-core processor and 32 gigabytes of RAM. To optimize image analysis, nuclear and cytoplasm segmentation parameters were interactively fine-tuned in multiple tissue samples with repeated standardized experiments. The results were examined for plausibility using the function ‘backward connection’. This function allows for visualization of the currently selected events and control of the appropriateness of the segmentation parameters used. The segmentation values with the most plausible results were then selected for the whole experiment (Supplementary Tables 1 and 2).

Regions with artifacts, debris and air bubbles were visually identified and manually excluded. The epithelial layer and lamina propria were manually outlined as subregions using the software function ‘create region of interest’ to survey both tissues separately.

### Nuclear segmentation

**Fig. 1:**
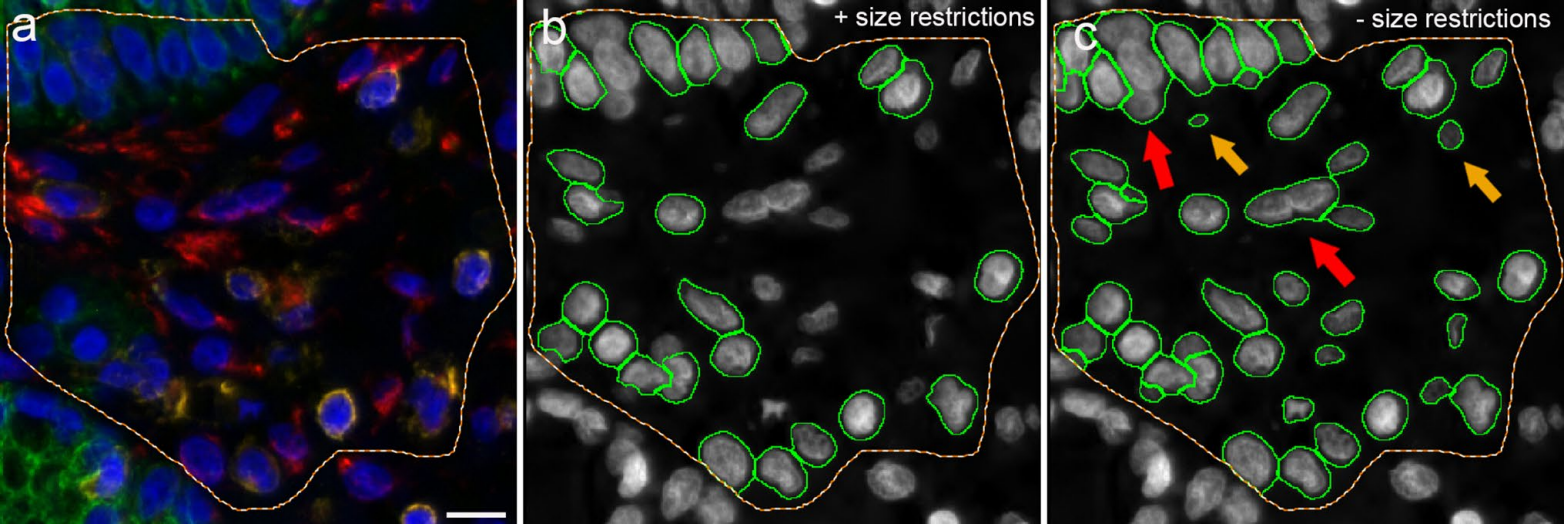
Nuclear segmentation. Four-channel fluorescence microscopy, scale bar 10 μm, × 40. Male patient with chronic rhinosinusitis without polyps. **a** Green pancytokeratin antibody, red vimentin antibody, yellow CD45 antibody combined with CD18 antibody and blue nuclear DAPI staining. The yellow line demarcates the examined tissue area. **b**, **c** Comparison after nuclear segmentation with and without the function ‘remove labels’ in DAPI channel. The nuclear segmentation was performed only in the area demarcated with a yellow line. ‘Remove labels’ was set to exclude nuclei smaller than 40 μm^2^ and larger than 100 µm^2^. Each green circle demarcates an identified nucleus, which corresponds with cells in (**a**). **b** With ‘remove labels’, 24 cells were identified. **c** Without ‘remove labels’, 46 cells were identified. The red arrows point at two nuclear conglomerates, the left upper and right lower arrows point at conglomerates, of 3 and 2 nuclei, respectively. Each conglomerate was falsely identified as 1 nucleus. The yellow arrows point at nuclei < 40 μm^2^. With use of the function ‘remove labels’, 53,591 cells were identified in the entire tissue sample

Nuclear segmentation was used to identify each individual nucleus and was performed in the DAPI channel (master channel). Several nuclear segmentation parameters were used (Supplementary Table 1). Nuclear dimensions were ensured to be precise by excluding an area size smaller than 40 µm^2^ and larger than 100 µm^2^ using the function ‘remove labels’. This function served to discard flawed events from being recognized as a nucleus, such as nuclear conglomerates or nuclei cut at a marginal sphere segment. The aim was the recognition of biologically plausible nuclei (Fig. 1).

### Cytoplasm segmentation

Cytoplasm segmentation was performed by applying a cell mask emanating from the nuclear borders for each channel. Sectioning thickness implies cytoplasmic immunostaining around and overlapping the cell nucleus. Therefore, the cell mask was allowed to grow outside as well as inside the nuclear margin with the function ‘use identified cell mask—outside and inside’. Starting from the nuclear margin, this function propagates into the periphery in predefined ‘growing steps’ within the appropriate channel and toward the center of the nucleus until either a sudden step in staining intensity is detected or the center of the nucleus is reached. Several additional cytoplasm segmentation parameters were used separately for each channel (Supplementary Table 2).

### Background calculation

After nuclear and cytoplasm segmentation, an area free of nuclei was analyzed in each tissue sample after setting the nuclear segmentation parameters to 0. After segmentation, multiple small events were recognized, corresponding to pixels of the analyzed area.

### Data analysis

All Raw Data were extracted into the SPSS 26.0 statistic package (SPSS Inc., Chicago, Illinois, USA). After normalization, a 30% random sample of all cells was chosen. For differentiation of positive and negative cells, the upper bound of the 95% confidence interval (CI) of the relative differences of the median values of the mean intensities of cells and background [(median intensity value of cell – median intensity value of the background)/median intensity value of cell] was calculated as the median cell/background ratio. A cell was then considered positive if its individual cell/background ratio was higher than the upper bound of the 95% CI of the median cell/background ratio. Then, cell populations with all possible combinations of the three antibodies were generated. The results were calculated as percentages (positive cell count per cell population/all cells).

For isotype controls, each channel’s mean intensity was compared with the mean intensity of each corresponding channel of the patient tissue using ROC analysis. To compensate for channel spillover, a single cytoplasmic-immunostained tissue sample was examined for positive cells in the remaining two cytoplasmic immunostainings.

## Results

### Patient population

The mean patient age (± SD) was 32 ± 13 years (range 18–57). Of the nine patients, seven were men. The mean (± SD) disease duration was 51 ± 42 months. Atopy and asthma were diagnosed in 2/9 patients. Hyposmia was reported by 4/9 patients. The mean (± SD) Lund–Mackay (Lund and Mackay 1993) score for the CRS patients was 12 ± 6 (range 3–20).

### Tissue area, cell count, cell density and nucleus size

Acquisition of a 10-mm^2^ tissue area required 90 min. To limit the illumination time of the fluorescent tissue samples, we preferred to limit the acquisition to parts of the slide. For one patient, we exploratively performed whole-slide acquisition. We observed no severe variations between whole- and partial-slide acquisition samples. However, this acted as an outlier for the scanned area and cell count. Therefore, a nonparametrical data analysis was performed. The median area scanned per patient was 4.5 mm^2^ (lower quartile 3.5 mm^2^ to upper quartile 6.8 mm^2^). A median of 14,785 cells (lower quartile 12,774 to upper quartile 20,100) was recognized per patient. The median cell density was 3486 cells/mm^2^ (lower quartile 3014 cells/mm^2^ to upper quartile 3952 cells/mm^2^). The median nucleus size was 64 µm^2^ (lower quartile 59 µm^2^ to upper quartile 67 µm^2^).

The epithelial area per patient (median 0.37 mm^2^; lower quartiles 0.3 mm^2^ to upper quartile 1.4 mm^2^) was smaller than the LP area (median 3.9 mm^2^; lower quartile 3.1 mm^2^ to upper quartile 5.5 mm^2^; Wilcoxon paired samples *p* = 0.008; Table 3). No significant differences in scanned area, cell count, cell density or nucleus size were observed between the patients with CRSsNP or CRSwNP and the controls (data not shown).

**Table 3:** Tissue area, cell count and cell density in epithelial layer and lamina propria for all patients

	Epithelial layer	Lamina propria	*p* value^a^
Tissue area (mm^2^)	0.8	5.6	0.008^b^
Cell count	3040	16,970	0.008^b^
Cell density (cells/mm^2^)	4022	3337	0.139^c^

^a^Wilcoxon Signed Ranks Test

^b^Based on negative ranks

^c^Based on positive ranks

### Cell populations

**Fig. 2:**
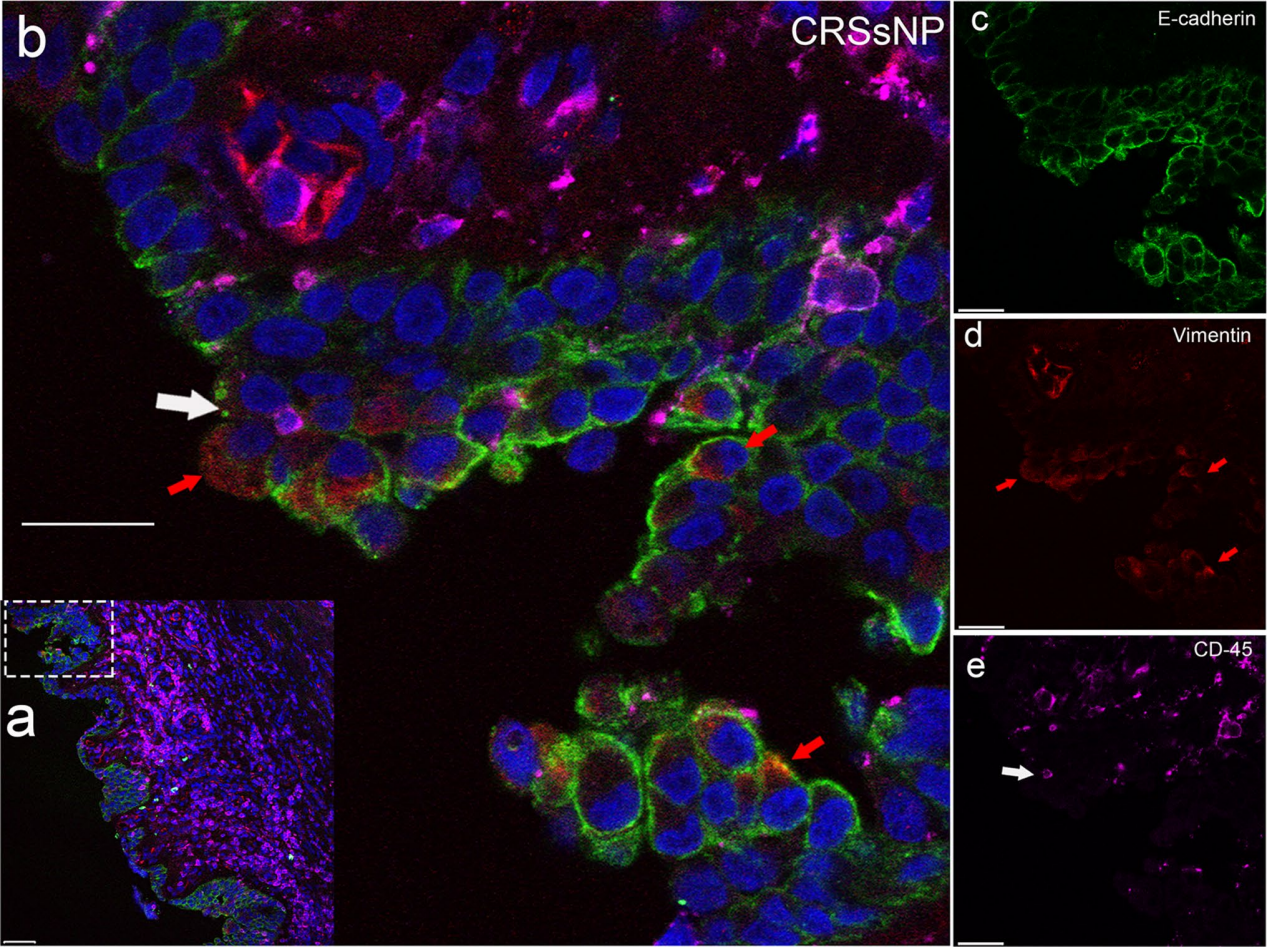
Coexpression of cytokeratin and vimentin. Four-channel fluorescence confocal microscopy, scale bar 20 μm, × 40. Male patient with chronic rhinosinusitis without polyps. **a** Overview of field of view. **b** Higher magnification of area (**a**) indicated by the box, showing a large amount of immune cells in the lamina propria. **c** Green: epithelial cell marker; **d** red: fibrocyte marker; **e** magenta: leukocyte marker. Blue: nuclear DAPI staining. The white arrow points at leukocytes invading the epithelium. Red arrows point at epithelial–mesenchymal transition (EMT) cells in the epithelium

The most common cell types were cytokeratin-single-positive (26%), vimentin-single-positive (13%) and CD45/CD18-single-positive with CD45/CD18–vimentin-double-positive cells (presumably immune cells; 29%). Triple-negative cells comprised 8%. Interestingly, 3% were cytokeratin–vimentin-double positive. The existence of cytokeratin–vimentin-double-positive cells was confirmed by confocal imaging (Fig. 2). A notable fraction of cells was cytokeratin–CD45/CD18-double positive (10%) or triple positive (11%).

### CRSsNP, CRSwNP and controls

Due to the small number of patients in this pilot study, the data were purely explorative. The difference in vimentin-single-positive cells of a median of 20% in the controls, 12% in CRSsNP and 7% in CRSwNP is striking. The number of CD45/CD18-single-positive cells in the patients with CRS was 3–6 times as high as that in the control patients, while the reverse trend emerged for the CD45/CD18–vimentin-double-positive cells (Table 4).

**Table 4:** Cell populations per diagnosis in epithelial layer and lamina propria

	Epithelial layer			Lamina propria			All patients^c^
Cell populations^a^ and comparison^b^	Control (A)	CRSsNP (B)	CRSwNP (C)	Control (A)	CRSsNP (B)	CRSwNP (C)	
Cytokeratin-single positive	55.8%	72.7%	72.0%	14.6%	22.2%	12.6%	26% ± 9%
		A	A	C	A, C		
Vimentin-single positive	0.8%	1.0%	0.3%	21.5%	13.7%	10.3%	13% ± 8%
	C	C		B, C	C		
CD45/CD18-single positive	0.6%	0.1%	0.9%	2.5%	6.7%	17.7%	6% ± 5%
	B		B		A	A, B	
CD45/CD18–vimentin-double positive	0.8%	0.2%	0.4%	44.5%	16.4%	30.3%	23% ± 14%
	B			B, C		B	
Cytokeratin–vimentin-double positive	0.4%	2.0%	1.1%	2.5%	4.1%	2.3%	3% ± 2%
		A, C			A, C		
Cytokeratin–CD45/CD18-double positive	29.3%	17.0%	18.6%	3.9%	11.0%	6.2%	10% ± 7%
	B, C				A, C	A	
Triple positive	11.2%	6.2%	5.2%	4.7%	15.4%	9.0%	11% ± 5%
	B, C				A, C	A	
Triple negative	1.1%	0.8%	1.5%	5.9%	10.6%	11.7%	8% ± 3%
			B		A	A, B	

a. Percentage of cell count in each cell population per all cells

b. Results were based on two-sided tests. For each significant pair, the key of the category with the smaller column proportion appeared in the category with the larger column proportion. Significance level for upper case letters (A, B, C) was 0.05. Tests were adjusted for all pairwise comparisons within a row of each innermost subtable using the Bonferroni correction

^c^Mean value ± standard deviation in both epithelial layer and lamina propria. For simplicity, decimal places were avoided

### Epithelial layer and lamina propria

Cell populations differed in the epithelial layer and in the LP (Table 4). In the epithelial layer, 69% of the scanned cells were cytokeratin-single positive compared to 19% in the LP (Wilcoxon paired samples *p* < 0.01). Accordingly, vimentin-single-positive cells (15%) were more abundant in the LP than in the epithelial layer (0.6%; Wilcoxon paired samples *p* < 0.01). Inflammatory cells, i.e., CD45/CD18-single-positive and CD45/CD18-vimentin-double-positive cells were more common in the LP (34%) than in the epithelial layer (1%; Wilcoxon paired samples *p* < 0.01).

### Tissue outline

**Fig. 3:**
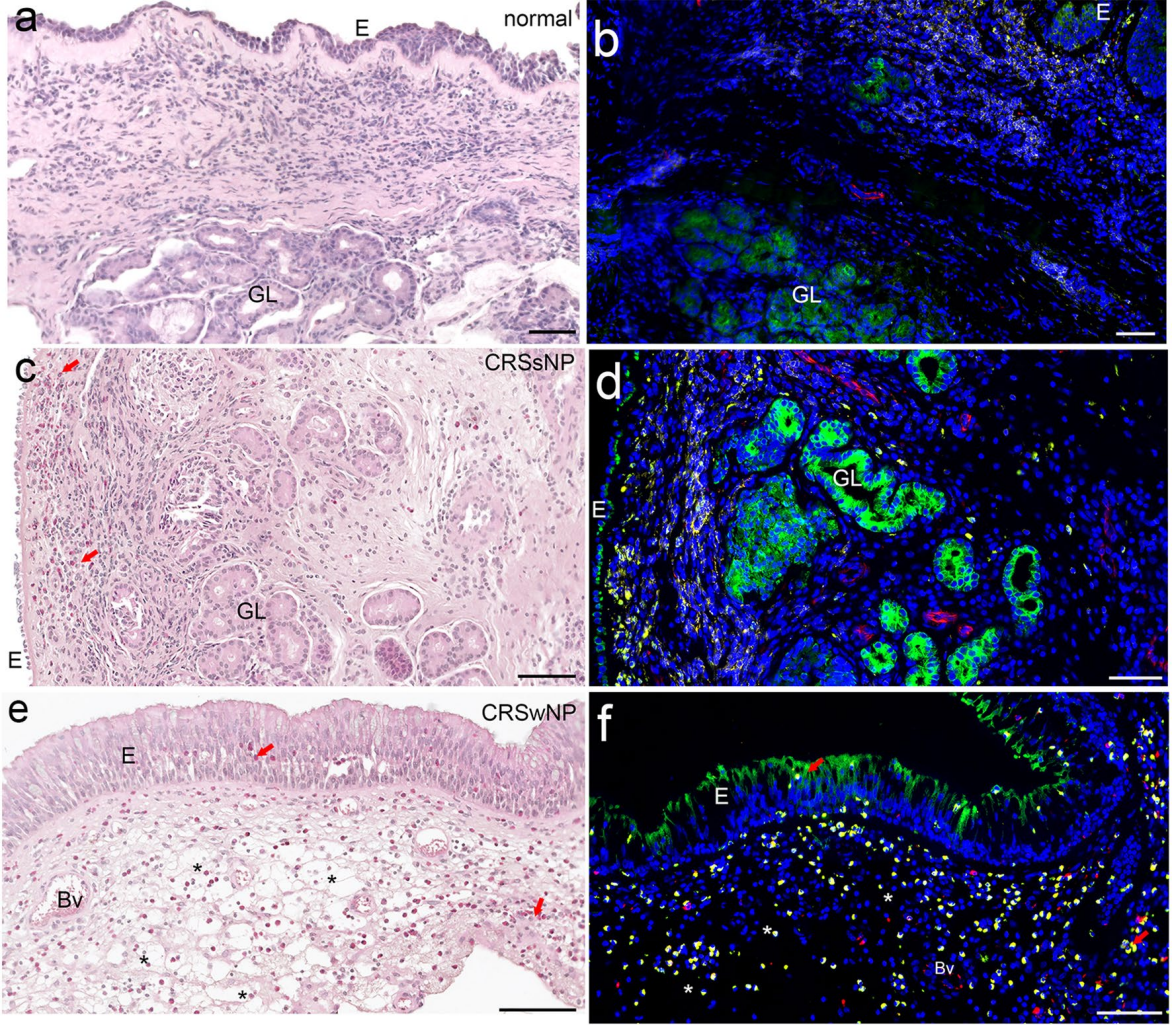
Chronic rhinosinusitis types and normal tissue; comparison of bright-field and fluorescent-immunolabeled sections. H.E. staining **(a, c, e)** and immunofluorescence staining **(b, d, f)** of control **(a, b)**, chronic rhinosinusitis without polyps (CRSsNP) **(c, d)** and chronic rhinosinusitis with polyps (CRSwNP) **(e, f)** tissue. Four-channel fluorescence represents nuclear DAPI staining (blue), cytokeratin (green), vimentin (red) and CD45 combined with CD18 (yellow). Scale bar: 100 μm

The epithelial layer presented as a pseudostratified columnar and partially ciliated respiratory epithelium covering a thick, vascular, glandular tissue layer (Fig. 3a). The LP presented as a compact layer; the luminal surface area of blood vessels was normal in size, and glands were arranged in distinct patches. IF staining exposed CD45-positive immune cells underneath the epithelial layer that were hardly distinguishable by H.E. staining. Typically, H.E. staining for the CRSsNP patients exposed eosinophilic cells that were identified as eosinophilic granulocytes (Fig. 3c). Blood vessels were partially dilated, and larger intracellular spaces were visible in the LP. The number of immune cells interfered with that was visible in IF images (Fig. 3d). CRSwNP often presented vastly dilated blood vessels and edema in the LP (Fig. 3e). Eosinophilic granulocytes were visually increased in all samples, and they were evenly distributed within the LP but also invaded the epithelial layer (Fig. 3e—red arrow in the E-layer), which was verified by IF (Fig. 3f). It was more difficult to identify edema in the IF images (Fig. 3f—asterisks) compared to the bright-field picture (Fig. 3e—asterisks).

### Immunostaining details

(a) Epithelial marker: Cytokeratin as well as E-cadherin-single-positive cells were mainly observed in the epithelial layer and in submucosal glands (Figs. 2, 3). The epithelial layer presented columnar cells with round to elliptic nuclei with intense intracytoplasmic staining. Confocal microscopy revealed that this intermediate filament was augmented at the cytoplasmic membrane and appeared to replenish the entire cytoplasm in epifluorescent images. Staining intensity seemingly increased in the outermost epithelial layer facing the outside lumen. This layer consisted of the most mature and stress-exposed cells. Furthermore, many dead and moribund cells were found here based on bright-field imaging. Autodigestive processes similar to proteolytic antigen retrieval techniques may favor the exposure of antibody epitopes. This may additionally allow for better access of antibodies compared to intact cells and contribute to this gradient in epithelial markers such as cytokeratin and E-cadherin. Occasionally, this resulted in acquired images with camera sensor oversaturation in some of these cells. The cytokeratin-positive epithelium of the seromucous glands pervaded the LP in the shape of convoluted tubes that intermingled with the rich capillary network embedded in the connective tissue.

(b) Mesothelial marker: Vimentin-single-positive cells mostly comprised fibrocytes forming the connective tissue in the LP with the classical spindle-shaped mesothelial cell body and equally shaped cell nucleus. Immunoreactivity was mostly confined to the soma of these cell types, and cell processes were not visible in IF images. Obviously, the endothelial cells were highly positive for anti-vimentin antibodies, outlining blood vessels in the LP and submucosa (Fig. 3b, d, f).

(c) Immune cell marker: A CD45/CD18-single-positive cell population dominated the LP. These cells represented lymphocytes, granulocytes and macrophages. Numerous small roundish cells with spherical nuclei showed only sparse cytoplasm and were typically considered to be lymphocytes. Lobulated nuclei indicated the presence of granulocytes, and the largest CD45/CD18-single-positive cells were likely macrophage-like cells. Some CD45/CD18 cells invaded the epithelial cell layer as far as the most superficial layer and appeared with equal morphology as in the LP or sent out small processes between epithelial cells. Most immune cells in the mucosa faced the most basal layer of the epithelium.

(d) Double-positive cells: CD45/CD18–vimentin-double-positive cells often presented an image overlay of immune cells and adjoining mesothelial cells, such as cell processes of fibrocytes, and projected overlays with the cell somata of endothelial cells or fibrocytes. Cytokeratin–vimentin-double-positive cells represented overlay artifacts with other neighboring cells as well as real EMT cells.

(e) Negative cells: Triple-negative cells were mostly fibrocytes with low immunoreactivity due to delicate processes and sparse cytoplasm. Cell masks of cytoplasmic staining detected by trained algorithms computed low mean intensities that dropped below the criteria set for positive immunoreactivity.

### Isotype controls and compensation of channel spillover

**Fig. 4:**
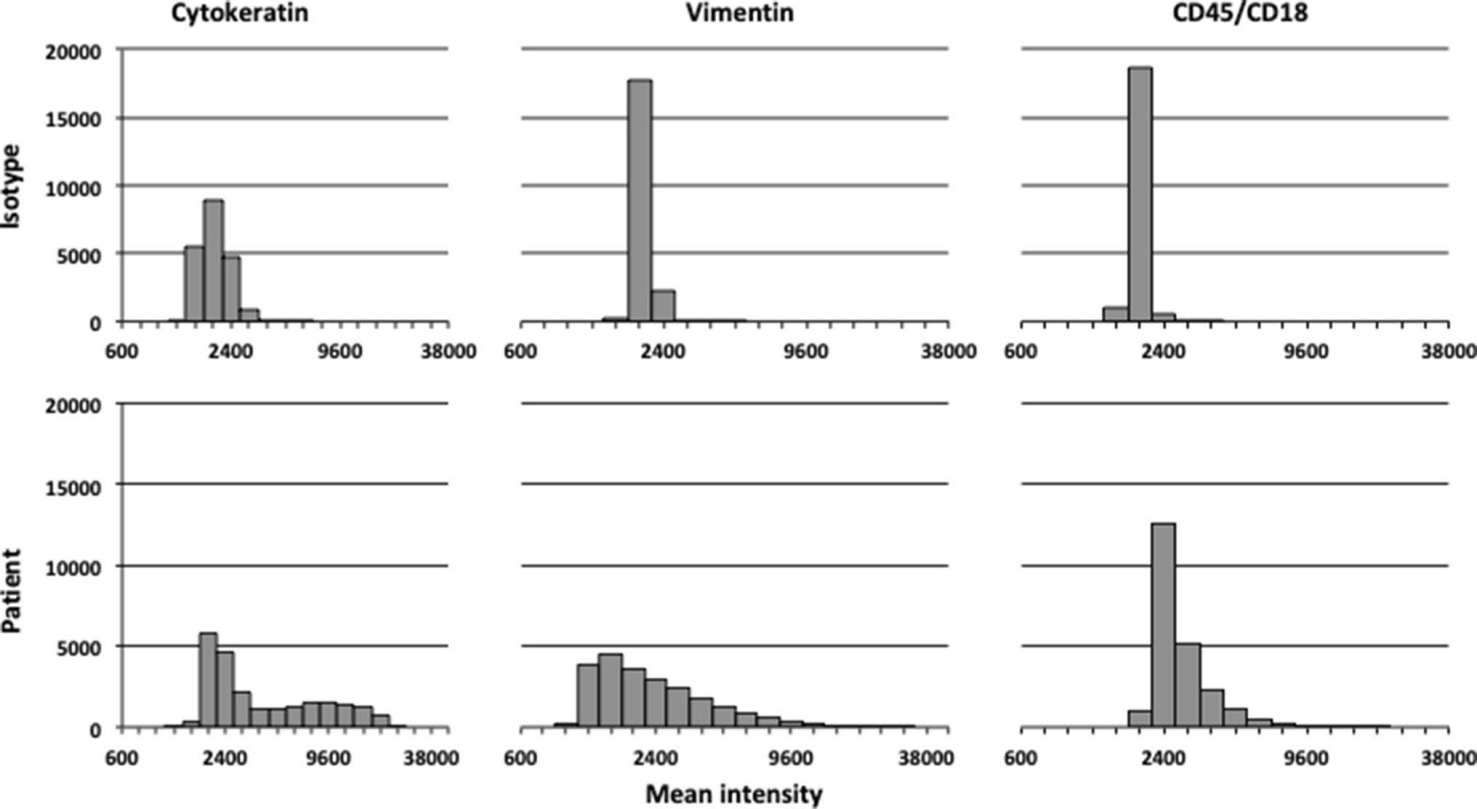
Comparison of histograms of fluorescence signals between isotype controls and patient’s tissue. Histograms of fluorescence signals after control incubation in the upper histograms (isotype controls) and specific fluorescence signals after test incubation in the lower histograms (patient’s tissue). Presentation of a random cell sample of all patients and their isotype controls. *X*-axis: mean intensity in logarithmic scale. *Y*-axis: cell count. The left histograms represented cytokeratin fluorescence signal, the middle histograms vimentin fluorescence signal and the right histograms CD45/CD18 fluorescence signal

Each channel’s fluorescence signals after control incubation (isotype controls) differed significantly from the corresponding channel’s specific fluorescence signals after test incubation (patient’s tissue) (Fig. 4). In isotype controls, approximately 95% of the cells were triple negative. Channel spillover between AF488, eFluor 570 and AF594 in all possible combinations was examined and found to be lower than 1%.

## Discussion

Given the possibilities that modern multicolor image analysis systems offer (Gokhale 2016; Blom et al. 2017), we established a four-channel IF microscopy technique for simultaneous detection of three antibodies and DAPI in FFPE tissue samples of the human upper airway mucosa. IMIC allows for quantification of cells in tissue samples. However, it is an elaborate process, and all steps from sample collection to statistical evaluation involve numerous possibilities for errors.

### Sampling site

In CRS, tissue samples were collected from the anterior ethmoidal region during FESS. This constitutes common practice in rhinology. While data for histopathologic tissue variation between the paranasal sinuses are sparse, specimens from the anterior ethmoidal region are widely used for description of CRS histopathology and are considered both patient specific and patient representative (Ganti et al. 2020; Marino et al. 2019; Polzehl et al. 2006; Snidvongs et al. 2012; Tomassen et al. 2016). In control patients, tissue samples were collected from resected inferior turbinate mucosa. These specimens are harvested during anterior turbinoplasty and are widely used from rhinologists for histopathologic examination of inferior turbinate hyperplasia (Polzehl et al. 2006; Van Zele et al. 2006). They are also considered patient specific and patient representative.

### Method development

In a first approach to simultaneously quantify different cell types, we used colorimetric immunohistochemistry of serial sections stained with different antibodies. Despite accurate alignment of the serial sections by modern systems (Jepsen et al. 2018), the alignment was not precise enough for coexpression quantitative analysis of biomarkers in individual cells. Multichannel fluorescence microscopy is better suited because fluorescence markers can be better separated than can the color deconvolution algorithms used for enzymatic staining. Moreover, the precision of localization is superior for immunofluorescent imaging, especially when directly labeled primary antibodies are available. On the contrary, identification of different layers, e.g., epithelial layer, is not always easy for immunofluorescent imaging. Indeed, serial sections enable the investigators to follow the course of different layers that is not always easy to identify along its 3D course within a specimen. Here, the extended depth of focus with autofocus and two 1.7-μm steps above and two below helped us to reflect the 3D tissue structure.

Antibody selection is a basic problem. Combinations of the primary and secondary antibodies used for upper airway mucosa samples resulted in unreliable staining and high background. The upper airway mucosa contains various proteolytic enzymes that can disrupt the binding of secondary to primary antibodies. In contrast, directly labeled antibodies provided reproducible and biologically plausible results. Unfortunately, the availability of directly labeled antibodies is limited. The directly labeled pancytokeratin and the directly labeled CD45 antibodies were chosen for the detection of epithelial cells (Hicks et al. 1995) and immune cells (Yu et al. 2002), respectively. The CD45 antibody used is the HI30 monoclonal antibody, which reacts with all isoforms of human CD45. Nevertheless, a significant amount of leukocytes was not stained (data not shown). To improve detection, we added an in-house-made direct-conjugated CD11b antibody, though this did not improve leukocyte detection and resulted in nonspecific background staining. A mixture of directly labeled antibodies against CD45/CD18 extended our ability to detect immune cells and provided more plausible results without nonspecific background staining. CD18 plays an important role in neutrophil transmigration (Fukuda and Schmid-Schonbein 2003). Specific detection of fibroblasts was particularly problematic. Despite the reported good specificity of S100A4 for fibrocytes (Cunningham et al. 2010), we observed nonspecific reactions, particularly in regions with a high mucus content. Moreover, pretreatment with 0.3 mg/ml dithiothreitol as a mucolytic agent (Chisholm et al. 2013) did not improve the results. We then replaced the S100A4 antibody with a directly labeled vimentin antibody; we did not observe any nonspecific staining of mucus with vimentin, but leukocytes cross-reacted (Lilienbaum et al. 1986). This is probably due to the common mesodermal origin of fibroblasts and blood-derived immune cells (Lysy et al. 2007; Ding and Morrison 2013). We finally chose the presence of vimentin and absence of CD45/CD18 to identify connective tissue cells (Bourin et al. 2013).

**Fig. 5:**
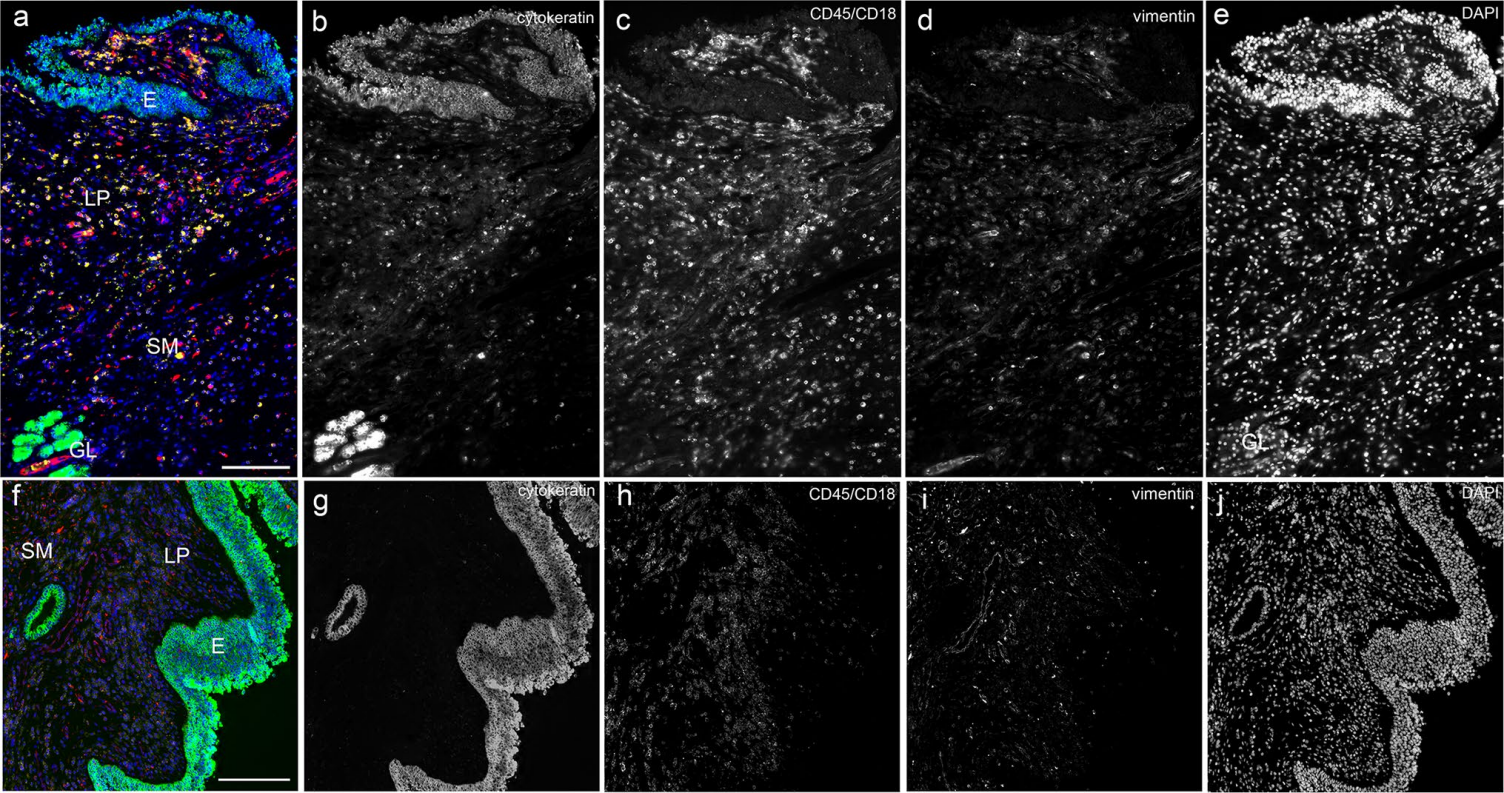
Autofluorescence reduction with the use of the quenching kit. Four-channel fluorescence microscopy, scale bar 200 μm, × 40. **a–e** Without quenching; **f–j** with quenching autofluorescence. Male patient with chronic rhinosinusitis without polyps. Green: pancytokeratin antibody, red: vimentin antibody and yellow: CD45 antibody combined with CD18 antibody. Autofluorescence is obvious in channels AF488 (**b**), AF594 (**c**) as diffuse background. eFluor 570 (**d**) and DAPI (**e**) mainly showed unspecific fluorescence inside blood vessels and DAPI in submucosal glands and epithelial layer as well. **f–j** With the use of the quenching kit, a good reduction of autofluorescence is obtained for all excitation and filter sets used. E: epithelial layer, LP: lamina propria, SM: submucosa, GL: submucosal glands

Autofluorescence is inherent fluorescence from a cell to which no stain or fluorochrome has been added (Wang and Hoffman 2017). It often occurs with aldehyde fixation or due to inherent native tissue components (collagen, elastin, high nicotinamide adenine dinucleotide content). Fluorescence background is also a component of FFPE tissue samples, which cannot be attributed to specific structures or cells. Most methods to reduce autofluorescence act primarily on lipofuscin granules and include “home brew” concoctions such as sodium borohydride and other ink-based products, which are essentially ineffective against aldehyde-induced autofluorescence. In contrast, the quenching kit used binds to hydrophilic compounds and effectively quenches endogenous autofluorescence, as used in recent studies (Davies et al. 2019). Autofluorescence was almost abolished using the quenching kit (Fig. 5).

Image acquisition was improved in several steps. In the first set of image acquisitions, we used 20 × magnification with an aperture (*A*) = 0.6, which yielded lower image quality for reliable identification of the cell nucleus and cytoplasm. In a second set of image acquisitions, we used a 40 × oil immersion objective with high aperture (*A* = 1.3); however, during unobserved image acquisition, contamination of the system with overflowing immersion oil caused problems. Finally, an air 40 × lens (*A* = 0.95) was a good compromise between image resolution and practicability of imaging with high throughput.

The PCO pixelfly CCD dynamic range camera recorded the emitted fluorescence signal in a 14-bit data range. In our first approach, we used the software TissueQuest (TissueGnostics), which provided data in 8 bits. To broaden and better differentiate the fluorescence signal between cells, we used the software StrataQuest, which provided data in 14 bits.

### Nuclear segmentation

In contrast to flow cytometry, in which individual cells are always completely recorded when they pass the photodetector, partially cut cells are present in tissue samples. Moreover, dense aggregation of cells within a tissue section hampers cell separation and detection. Seemingly, cell agglomeration results from projection of the 3D distribution of small cells in a 5-µm-thick tissue slice. As the cell nuclei of the examined cell lineages differ in size and shape (Skinner and Johnson 2017), nuclear segmentation can influence the probability with which the cells are included in the analysis and cause serious systematic bias. This possible source of bias was extensively examined using the software function ‘backward connection’.

Nevertheless, potential bias should be mentioned. The false recognition of nuclei conglomerates as one large nucleus, even in a low non-significant frequency, cannot be excluded. Furthermore, to discard flawed events from being identified as a nucleus, areas smaller than 40 µm^2^ were excluded from the analysis. As a ‘side effect’, cells with a nuclei cut at a marginal sphere segment were also excluded from the analysis. This may have resulted in oversampling cells with nuclei size larger than 40 µm^2^. This implied that the trade-off between biologically plausible nuclei, flawed events and under- or oversampled cell populations was difficult to avoid.

### Cytoplasm segmentation

In the eFluor 570 channel, the ‘max growing steps’ value was set higher than in AF488 and AF594 (4.0 compared to 2.0). This channel comprises connective tissue cells, including fibroblasts, which frequently present an elongated cell form. Thus, the segmentation parameters of the eFluor 570 channel were targeted to further search for the examined cytoplasm when compared to the other channels. Since the vimentin content is often low in the direct vicinity of the nuclear membrane, this skipping of a growing step helped to capture real cell-allocated immunoreactivity. Moreover, in tissue slices, neighboring cells can partially overlap and simulate coexpression of biomarkers by projecting different focal planes in the *z*-axis. Using a slice thickness of 5 µm reduced this risk of bias.

### Cell populations

The characterization of a cell as positive was based on the rationale that cell-specific immunostaining should be significantly higher than its background. In the software, no background subtraction was available. For this reason, we developed the cell/background ratio, which was 1.44, 2.01 and 1.18 in the AF488, eFluor 570 and AF594 channels, respectively. Although characterization of single-cell populations was unproblematic, certain irregularities emerged due to the low AF594 ratio. This resulted in classifying cells as double or triple positive, and the mean intensity of the simultaneous examined antibodies differed significantly. This was obvious in cytokeratin–CD45/CD18-double-positive cells. In these cells, the median value of the mean intensity of cytokeratin was ten times higher than that of CD45/CD18. This indicated that most of the cytokeratin–CD45/CD18-double-positive cells were actually cytokeratin-single-positive cells.

**Fig. 6:**
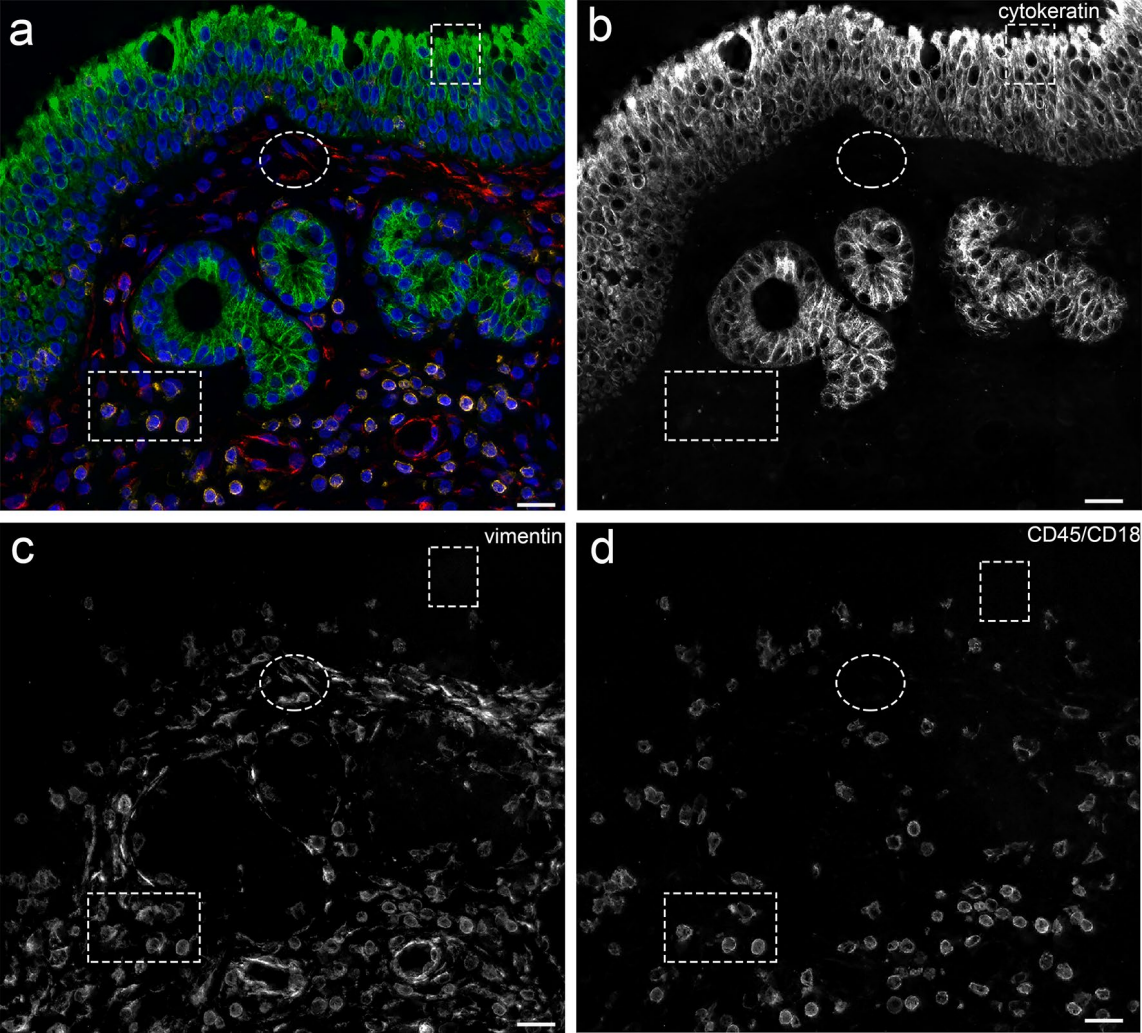
Cell populations in four-channel fluorescence microscopy. **a** Four-channel fluorescence microscopy, scale bar 20 μm, × 40. **b** AF488 channel. **c** eFluor 570 channel. **d** AF594 channel. **a–d** Male patient with chronic rhinosinusitis without polyps. Green: pancytokeratin antibody, red: vimentin antibody and yellow: CD45 antibody combined with CD18 antibody. The small square frame indicates the position of a *cytokeratin-single-positive epithelial cell*. The elliptic framed area points at a *vimentin-single-positive fibroblast*. The rectangular area shows a *CD45/CD18–vimentin-double-positive leukocyte*

Cells positive for cytokeratin and negative for vimentin and CD45/CD18 (cytokeratin-single-positive cells) were considered epithelial cells in the epithelial layer and glandular cells in the LP. The pancytokeratin antibody recognizes human cytokeratin 4, 5, 6, 8, 10, 13 and 18. This combination should cover all epithelial cells recognized in the nasal mucosal membrane (Stosiek et al. 1992; Liu et al. 2017; Hicks et al. 1995). As expected, cytokeratin-single-positive cells were more abundant in the epithelial layer than in the LP. Data on cytokeratin expression in the glands of human nasal nondiseased mucosa are sparse. In our study, glands were adequately stained (Fig. 6a). We noted a higher percentage of glandular cells in CRSsNP (Table 4), which was in line with relevant reports (Berger et al. 2002; Schleimer 2017).

Cells positive for vimentin and negative for cytokeratin and CD45/CD18 (vimentin-single-positive cells) were considered connective tissue and endothelial cells (Fig. 6a) (Lan et al. 2018). Connective tissue cells in the upper airway mucosa include fibroblasts and fibrocytes. Vimentin is expressed in fibrocytes, fibroblasts, endothelial cells, and leukocytes, including macrophages, neutrophils, lymphocytes (Satelli and Li 2011; Fuchs and Weber 1994; Steinert et al. 1993; McKeon et al. 1986; Steinert 1993; Crystal et al. 2008; Lilienbaum et al. 1986; Lan et al. 2018), and blood platelets (Ahmed et al. 2009). In our study, leukocytes were filtered out from the vimentin-single-positive cell population, as this population was negative for CD45/CD18. This cell population included endothelial cells in the endothelium. Since endothelial cells were less frequently observed (Fig. 6a), the vimentin-single-positive cell population included mainly connective tissue cells. We observed a higher percentage of vimentin-single-positive cells in controls, which was contradictory to the data in the literature (Carroll et al. 2016). However, this might be attributed to the small number of patients in this pilot study.

Cells positive for CD45/CD18 and negative for cytokeratin and vimentin (CD45/CD18-single-positive cells) as well as cells positive for CD45/CD18 and vimentin and negative for cytokeratin (CD45/CD18–vimentin-double-positive cells) were considered leukocytes (Fig. 6a). Studies have reported that CD45 identifies eosinophils (Lee et al. 2012; Blaylock et al. 2003), basophils (Han et al. 2008), mast cells (Chisholm et al. 2015), macrophages (Pilling et al. 2009), neutrophils, lymphocytes and monocytes (Yu et al. 2002). Studies have also reported that CD18 preferentially binds to neutrophils (Fukuda and Schmid-Schonbein 2003). Multiple antibodies have been used in leukocyte identification. It cannot be ruled out that the CD45/CD18 combination did not recognize all leukocytes. Our results revealed higher leukocyte percentages in the LP of CRSsNP and CRSwNP patients than in controls, which is in line with recent reports (Fokkens et al. 2020). The reverse trend was observed for CD45/CD18–vimentin-double-positive cells (Table 4). However, due to the low cell/background ratio of CD45/CD18, it was unclear which fraction of this cell population corresponded to immune cells and which fraction to connective tissue cells.

Cells positive for cytokeratin and vimentin and negative for CD45/CD18 (cytokeratin–vimentin-double-positive cells), located in the epithelium, could be cells undergoing the epithelial–mesenchymal transition (EMT) (Li et al. 2019; Yan et al. 2019) (Fig. 2). EMT cells were 3–5 times more abundant in patients with CRS than in control patients (Table 4). Schleimer supported the presence of chronic EMT in CRS (Schleimer 2017). Cytokeratin–vimentin-double-positive cells were also observed in the LP (Table 4). Specific examinations of these cells revealed that the vast majority were glandular cells neighboring connective tissue cells. The parameter ‘max growing steps’ was set higher for the eFluor 570 channel to identify elongated connective tissue cells. This resulted in the identification of connective tissue cells but also in the false characterization of some glandular cells as cytokeratin–vimentin-double-positive cells. In the epithelial layer, the cytokeratin–vimentin-double-positive cells were not in proximity with any connective tissue cells.

Cells negative for all three antibodies comprised 8% of all scanned cells. Triple-negative cells were mostly fibrocytes with low immunoreactivity due to a sparse cytoplasm and were mainly found in the LP (Table 4). Most of the triple-negative cells were weakly stained vimentin-single-positive cells corresponding to connective tissue cells.

Cells positive for cytokeratin and CD45/CD18 and negative for vimentin (cytokeratin–CD45/CD18-double-positive cells) and cells positive for all 3 antibodies (triple-positive cells) were considered artifacts as a result of cytoplasm and cell mask overlap. Simultaneous expression of cytokeratin and CD45/CD18 and of binding of all three antibodies in cells is not known. These cells accounted for approximately 10% each. The low cell/background ratio of CD45/CD18 might have influenced the results. The vast majority of cytokeratin–CD45/CD18-double-positive cells were actually cytokeratin-single-positive cells. This observation could be supported by the high percentage of this cell population in the epithelial layer (Table 4). Similarly, the vast majority of triple-positive cells were actually cytokeratin–vimentin-double-positive cells.

### Clinical significance

We intended to provide the first data for the simultaneous distribution of epithelial cells, connective tissue cells and immune cells in healthy and diseased human upper airway mucosa. This study was designed to investigate whether IMIC provides such data. While IMIC fairly succeeded in providing quantitative data of the above-mentioned cell types, such data were purely informative and lack clinical significance. The small number of patients did not allow significant conclusions for CRS pathophysiology. It is unlikely that examination of higher number of patients would change the purely informative character of the examined cell lineages in CRS. Nevertheless, the potential applications of IMIC in more clinically relevant areas of research may compensate for this study’s deficits in clinical significance.

### Future aspects

The potential applications of IMIC may include characterization of multiple cell types in CRS and cancer tissue, still with uncertain clinical applications. Current treatment regimens are based on the clinical classification into CRSsNP and CRSwNP. However, accumulating evidence suggests that CRS is a heterogeneous group of disorders with distinct pathogenic mechanisms (Cao et al. 2019). This heterogeneity influences treatment response. Wen and coauthors reported that increased neutrophilia in nasal polyps reduced the response to corticosteroids. Oral prednisone was found to shrink eosinophilic nasal polyps, whereas it failed to influence neutrophilic inflammation (Wen et al. 2012). Several studies reported the necessity of tissue eosinophilic quantification and grouping of pathogenic mechanisms into endotypes (Vlaminck et al. 2014; Soler et al. 2016; Akdis et al. 2013). It would be interesting to investigate whether data about the simultaneous distribution of cells such as neutrophils, eosinophils and T helper cells in solid tissue would remain purely informative or contribute to further classification of endotypes in CRS and alter treatment regimens.

In cancer, advances in immune-oncology have undoubtedly changed the standard of care for many different types of cancers. Complex interactions between immune cells and cancer cells in the tumor microenvironment can impact on clinical response to immunotherapy. Thus, profiling the immune contexture of tumor microenvironment may help to identify prognostic and predictive biomarkers of response to immunotherapy. Vassilakopoulou and coauthors reported that increased density of tumor-infiltrating lymphocytes favorable affects outcome in laryngeal squamous cell carcinoma. Such markers could be useful to predict individual response to immune checkpoint inhibitors (Vassilakopoulou et al. 2016). IMIC may allow more accurate characterization of the tumor microenvironment through distribution of epithelial and immune cells in tumor nodes and adjacent LP. Potential clinical applicability remains to be examined and unraveled.

## Conclusions

We provide the first relative distribution of epithelial, connective tissue and immune cells in FFPE human upper airway tissue samples. The analysis was based on IMIC. Multiple direct-conjugated antibodies were used for simultaneous coexpression detection. Isotype controls were used as negative controls. We developed a measurement to distinguish positive from negative cells based on cell-specific immunostaining intensity and background to cope with investigator-dependent issues. Analysis of the epithelial layer and lamina propria supported the interpretation of the results. The results were purely informative. The small number of patients did not allow important conclusions for CRS. It is unlikely that higher number of patients would change the purely informative character of these cell lineages in CRS. Nevertheless, IMIC fairly succeeded in providing quantitative data for simultaneous distribution of these cell lineages. Future studies may focus on the distribution and coexpression patterns of different immune cells in FFPE CRS and/or cancer tissue samples. Such data could support personalized treatment and evaluation of disease prognosis.

## Electronic supplementary material

Below is the link to the electronic supplementary material.

Supplementary file1 (DOCX 87 KB)

## Data Availability

Not applicable.
