# Supplementary material for: Characterization of epithelial cells, connective tissue cells and immune cells in human upper airway mucosa by immunofluorescence multichannel image cytometry: a pilot study
Source: Histochem Cell Biol. 2020 Nov 29;155(3):405–21. doi: 10.1007/s00418-020-01945-y (PMC8021535; doi:10.1007/s00418-020-01945-y)
Supplement: Supplementary file 1 — Supplementary file1 (DOCX 87 KB) [file 418_2020_1945_MOESM1_ESM.docx]

# Supplementary Material

## Supplementary Tables

### Supplementary Table 1. Parameters for nuclear segmentation used in the image analysis

| **Parameters for master channel DAPI** | **Value used in the study** | **Minimal value** | **Maximal value** |
| --- | --- | --- | --- |
| Nuclei size^a^ | 14 | 1 | 50 |
| Remove small-size objects^a^ | 1 | 1 | 9 |
| Remove weakly stained objects^a^ | 1 | 1 | 9 |
| Automatic Background Threshold^b^ | No | - | - |
| -Manual Threshold | 1500 | 0 | 65534 |
| Post Processing Order | Remove, Merge | Merge, Remove | Remove, Merge |
| Remove Labels | Yes | - | - |
| -Smaller Than | 40 μm^2^ | 0 | 123590.64 μm^2^ |
| -Larger Than | 100 μm^2^ | 0 | 123590.64 μm^2^ |
| -Weaker Than | Do not use | -1 | 65536 |
| -Stronger Than | Do not use | -1 | 65536 |
| Use Merging Rules^c^ | No | - | - |
| -Max Combined Area^d^ | - | 0 | 1920000 |
| -Max Involved Compactness | - | 0 | 1 |
| -Group Max | - | 2 | 4 |
| -Min Resulted Compactness | - | 0 | 1 |
| Use Border Input^e^ | No | - | - |
| -Border size^f^ | - | 1 | 1000 |
| -Border Option^g^ | - | - | - |

a. Arbitrary unit reflecting small and large nuclei, small-size objects and weakly-stained objects.

b. It aimed to discriminate background from specific cell objects. It enabled to define a grey value in the 16-bit range and covered 65.000 shades of gray. This value determined highest unspecific signal intensity and could be estimated by analyzing grey levels at areas without tissue using the software’s pixel inspector with the mouse cursor. All gray level values below the defined background threshold were excluded from the analysis.

c. This parameter searched for and merged groups of objects that made a round shape when combined. This could be useful in case of undersegmentation, when a round nucleus might be detected as 2 separate objects. We did not use this function in our study.

d. Arbitrary unit reflecting small and large combined areas.

e. If checked, a border for each FOV will be created before applying the analysis. This option helped for a better segmentation of nuclei that were at the border of the FOV and prevented the splitting of the nuclei at the FOV’s border.

f. Arbitrary unit reflecting small and large border size. It represented the size of the border added to a FOV.

g. This option should be used when a neighboring image does not exist. Four options were included. 1) Fill with black: border pixels will be set to value “0” (black). 2) Fill with white: border pixels will be set to value “65534” (white). 3) Copy last pixels: fills added border with the last pixels on border. 4) Mirror: copies pixels from the opposite side of the image.

### Supplementary Table 2. Parameters for cytoplasm segmentation used in the image analysis

| **Parameters for non-master channels** | **AF488 (cytokeratin)** | **eFluor570 (vimentin)** | **AF594** **(CD45/CD18)** |
| --- | --- | --- | --- |
| Use Ring Mask^a^ | No | No | No |
| -Interior Radius | - | - | - |
| -Exterior Radius | - | - | - |
| Use Identified Cell Mask^b^ | Outside & Inside | Outside & Inside | Outside & Inside |
| -Max Growing Steps^c^ | 2.00 μm | 4.00 μm | 2.00 μm |
| -Skip Steps^d^ | 1.00 μm | 1.00 μm | 1.00 μm |
| Use Nuclei Mask^e^ | No | No | No |
| Automatic Background Threshold^f^ | No | No | No |
| -Manual Threshold | 0 | 0 | 0 |

a. If selected, a ring mask was created around the objects detected in the master channel and included in the segmentation result. The interior and exterior radius allowed the examiner to define in which direction (inwards, outwards or both) and to what extent (from minus 666.67 μm to plus 666.67 μm) this ring should be created. The value represented the number of pixel layers. This ring construction stopped when the growing environment of a neighboring cell was reached (e.g. when cells touched each other). The value for exterior radius represented the maximum growth. The interior radius ring growth process stopped when the entire nucleus was covered.

b. It used the nuclei as seeds, e.g. starting point of image analysis and cell recognition. This function searched for specific staining pattern in the other channels of one FOV and causes binary objects to grow along those specific structures. Either ‘outside’ or ‘outside & inside’ could be used. By choosing ‘outside & inside’, growing was performed on the identified cell mask and might grow inside the nuclei mask if these two masks overlap. By choosing ‘outside’, growing was performed on the identified cell mask, but it would not grow inside the nucleus, even if there was staining in the nucleus.

c. It might limit this growth process with values from 0 μm to 333 μm.

d. In order to reach the marker staining, some additional growing steps might be required. In this case, the growing process would not start immediately at the nucleus border, but outside, at a distance specified by the function ‘skip steps’ with values from 0 μm to 333 μm.

e. If selected, the area of binary objects in the nucleus mask was also used as measurement mask for all other channels.

f. Similar as in nuclear segmentation parameters.
